# Zinc Sequestration: Arming Phagocyte Defense against Fungal Attack

**DOI:** 10.1371/journal.ppat.1003815

**Published:** 2013-12-26

**Authors:** Kavitha Subramanian Vignesh, Julio A. Landero Figueroa, Aleksey Porollo, Joseph A. Caruso, George S. Deepe

**Affiliations:** 1 Department of Molecular Genetics, Biochemistry, Microbiology and Immunology, University of Cincinnati, Cincinnati, Ohio, United States of America; 2 Division of Infectious Diseases, College of Medicine, University of Cincinnati, Cincinnati, Ohio, United States of America; 3 University of Cincinnati/Agilent Technologies Metallomics Center of the Americas, Department of Chemistry, University of Cincinnati, Cincinnati, Ohio, United States of America; 4 Divisions of Rheumatology and Biomedical Informatics, Cincinnati Children's Hospital Medical Center, Cincinnati, Ohio, United States of America; 5 Veterans Affairs Hospital, Cincinnati, Ohio, United States of America; Duke University Medical Center, United States of America

## Introduction

The innate immune system employs various defense mechanisms to combat invading microbes. From a pathogen perspective, access to adequate nutrition is one of the fundamental requirements for survival within the host. The ability to counter microbial survival by restricting basic elements of growth, extending from amino acids to sugars and metals, is referred to as nutritional immunity [1]. The mechanisms of Zn acquisition, transport, and storage have been investigated in both prokaryotic and eukaryotic systems. In this review, the total amount of zinc regardless of its chemical form will be referred to as Zn, and the labile fraction as Zn^2+^. From an immunological perspective, the primary focus has been on the impact of Zn regulation on the numbers and function of lymphocytes and phagocytes and their correlation with susceptibility to infections, but a dissection of the molecular details in these processes has been lacking. More recently, understanding the Zn modulatory mechanisms and how they drive host-pathogen interactions at the molecular level has been a subject of intense scrutiny. This review will accentuate existing and novel insights into the roles of Zn in nutritional immunity and in phagocyte defenses against fungi.

## Zinc Takes Center Stage: A Common Requisite in Host-Pathogen Interactions

Regulation of Zn homeostasis is essential for several host functions at multiple levels: i) for cellular processes including, but not limited to, transcription, translation, catalysis, and cell division; ii) for countering Zn^2+^ deficiency or excess; and iii) for immunomodulatory responses in host-pathogen interactions. An estimated 10% or 2,800 proteins in the human genome are Zn-dependent, implying a critical role for this metal in biological functions [2]. In the immune system, Zn regulation is of paramount importance as the development and function of innate and adaptive arms of immunity are influenced by this metal [3]. Zn homeostasis established by a balance in Zn^2+^ flux, intracellular distribution, and storage impacts phagocytosis, leukocyte recruitment, cytokine production, glycolysis, and oxidation triggered in response to immune signals. Aberrant Zn regulation in the circulation or in cells mitigates robust immune activation and leads to suboptimal host defenses. For example, Zn deficiency in humans with the genetic disorder acrodermatitis enteropathica is caused by Zn malabsorption and characterized by increased susceptibility to infections. An excess of Zn^2+^ diminishes T cell mitogenic responses [4]. Thus, an intact immune response requires strict Zn^2+^ regulation.

The fundamental requirement of Zn^2+^ for the function of several enzymes, transcription factors, and structural proteins [5] is evident not only in mammals but also in bacteria and fungi [6], in principal, due to the redox-inert property of this metal [7]. Zn^2+^ enhances the synthesis of toxic secondary metabolites such as *Aspergillus flavus* mycotoxins that inhibit phagocytosis and cytotoxicity of T cells [8]–[10]. Zn^2+^-dependent superoxide dismutases (SODs) produced by *Cryptococcus neoformans, Histoplasma capsulatum*, and *Candida albicans* are critical for scavenging superoxide radicals produced by phagocytes [11]–[13]. These factors underscore the significance of Zn acquisition and distribution for fungal pathogenesis and survival within the host. Thus, the struggle for Zn^2+^ between host and pathogen impacts survival of the invader and defense by the immune system.

## Zinc Acquisition Strategies: Host versus Fungi

**Figure 1:**
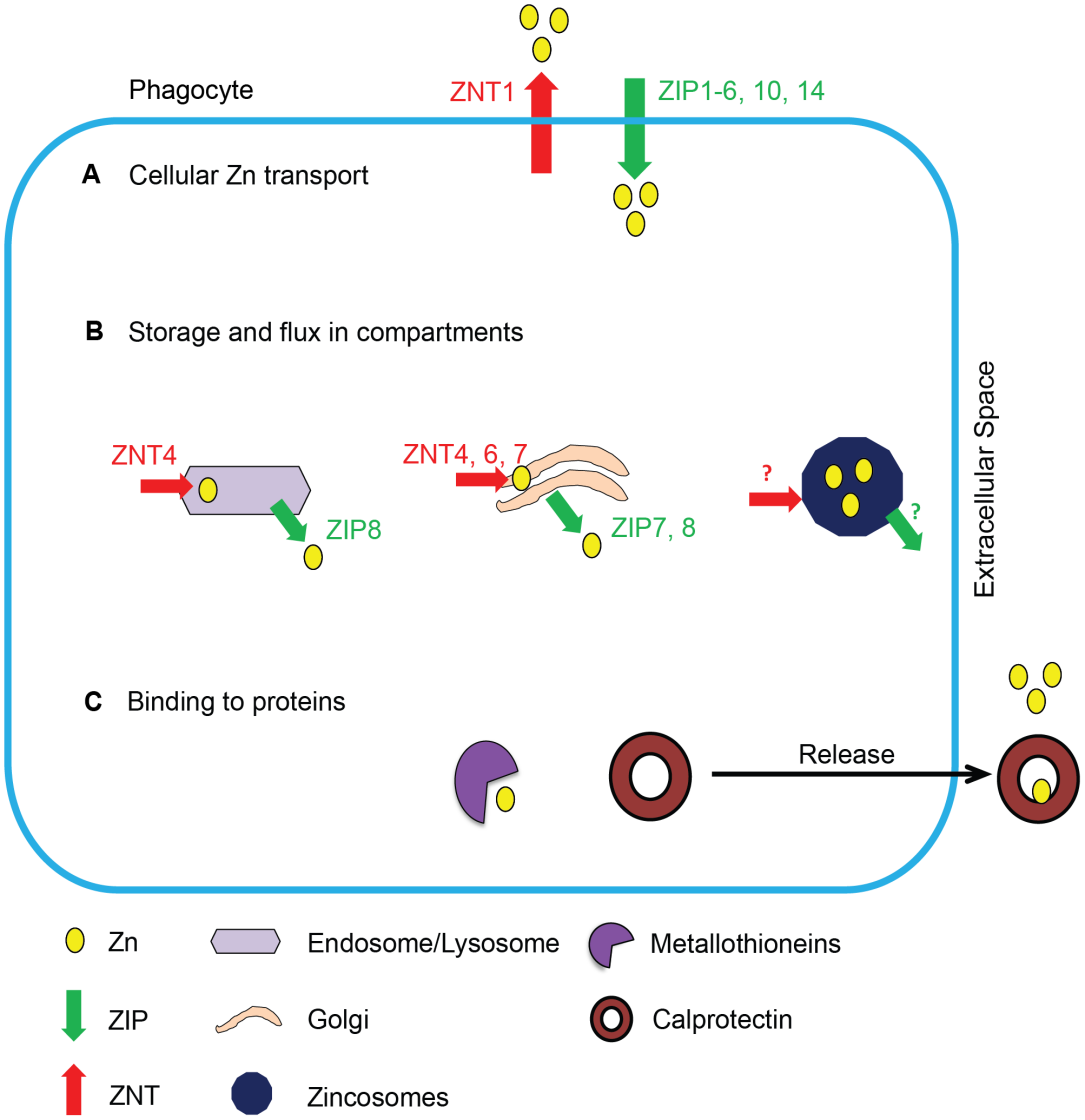
Schematic of Zn regulation in phagocytes. Mechanisms of Zn regulation in phagocytes, grouped into three categories: Zn^2+^ transport, storage, and binding. (**A**) Zn^2+^ transport across the cell membrane is mediated by ZIPs and ZNTs. (**B**) Intracellular Zn^2+^ is transported into and stored in organelles such as endosomes, lysosomes, Golgi, and zincosomes by various transporters represented in the figure; the transporters that mediate Zn^2+^ flux across zincosomes have not been identified. (**C**) Zn^2+^ is bound and sequestered by intracellular or secreted metal binding proteins such as MTs and calprotectin.

The immune system maintains Zn equilibrium via transporters, storage, and binding mechanisms **(** **Figure 1** **)**. While lower eukaryotes such as fungi possess fewer Zn^2+^ transporters [14], mammals have 24 transporters, called ZIPs (*Slc39a*, importers) and ZNTs (*Slc30a*, exporters). Some transporters manifest a ubiquitous expression pattern in several host cells, and others exhibit tissue specificity and function irreplaceably in Zn^2+^ transport. For example, *Slc30a1*is widely expressed in >12 organs, while *Slc39a4* expression is restricted to the small intestine and kidney and is absolutely essential for dietary Zn absorption [15]. Spatial organization of the transporters regulates Zn^2+^ in the cytosol and intracellular compartments including Golgi, mitochondria, and zincosomes that are a source of exchangeable metal during deficiency [16]. The remarkable complexity in Zn^2+^ transporters reflects the need for strict homeostasis and a regulatory system that responds to different biological stimuli in an organelle-, cell-, and tissue-specific manner. For example, interleukin-6 induces Zn^2+^ import via ZIP14 in hepatocytes [17], while granulocyte macrophage-colony stimulating factor (GM-CSF) triggers Zn^2+^ uptake via ZIP2 in macrophages [18]. The dependence of mammals on dietary sources for the metal implies the need for mechanisms that efficiently acquire Zn^2+^ and maintain regulated distribution in organ systems. Metallothioneins (MTs) comprise a class of metal binding proteins that regulate Zn^2+^ and prevent intoxication. MTs bind Zn^2+^ with picomolar affinity through seven binding sites, one of which is more readily exchangeable, and interactions with glutathione, ATP, or GTP mediate Zn^2+^ release [19]. These properties facilitate a controlled exchange mechanism in infected phagocytes, where Zn^2+^ access to the microorganism needs to be restricted. Thus, phagocytes possess manifold mechanisms to manipulate Zn resources during infection.

To establish infection, fungi must adapt to limited nutrient availability upon encounter with the host. Upon phagocytosis, *C. albicans* triggers a transcriptional response signature reflecting a state of nutrient deprivation within macrophages [20]. For pathogenic fungi, gaining entry into the host is associated with a transition from a possibly Zn^2+^-sufficient external environment [21] to a lower Zn^2+^-containing milieu. Similarly, for opportunistic fungi such as *C. albicans*, the shift from a commensal to a pathogenic state may be accompanied by a dramatic paucity in Zn^2+^, primarily due to Zn^2+^ restriction in the extracellular environment [22]. *Histoplasma capsulatum, Cryptococcus neoformans*, and *Blastomyces dermatitidis* thrive in soil containing 30–350 µM bioavailable Zn^2+^ [23]. Within macrophages, they are confronted with an environment containing only picomolar quantities of freely exchangeable Zn^2+^ [7]. To thwart host defenses and establish infection, fungi must exert strategies to sense and respond to metal scarcity caused by sequestration into intracellular niches or binding to host proteins via high affinity interactions. Mechanisms responding to Zn^2+^ availability in fungi include proteins that are directly affected by the presence or absence of Zn^2+^. For instance, Zn^2+^ inhibits DNA-binding activity of the Zn-responsive activator protein, Zap1p; however, a limiting milieu leads to transcription of Zap1p-dependent Zn^2+^ acquisition machinery. The upregulation of ZRT1 and ZRT2 transporters by Zap1p under a Zn^2+^-deficient state is critical, as the absence of these importers diminishes fungal pathogenicity [14], [24]. In *Cryptococcus gattii*, an ortholog of ZAP1 is upregulated by Zn^2+^ deficiency. Genetic deletion of ZAP1 impairs growth in a Zn^2+^-limiting environment, and mice infected with ZAP1 mutant yeasts exhibit increased survival [25]. These findings emphasize a role for Zn^2+^ regulation in fungal virulence.

Although extracellular fungi do not directly compete for the pool of Zn^2+^ within cells, they must secure the metal from a restricted environment in infected tissue. *A. fumigatus* possesses ZrfA and ZrfB analogous to Zrt1p and Zrt2p that facilitate Zn^2+^ uptake in a low-pH environment during deficiency [26]. In a specialized mechanism described as the “zincophore system,” *C. albicans* hyphae sequester host Zn^2+^ by secretion of pH-regulated antigen-1, which reassociates with Zrt1p for subsequent import [24]. Thus, multiple Zn^2+^ acquisition strategies in fungi collectively diminish vulnerability to host immunity. To establish virulence *in vivo*, these factors must contribute persistently to cope with metal scarcity induced by the immune system.

## Host Zinc Pool: Restricted Access

Microbes are extremely sensitive to metal availability, and phagocytes have mastered mechanisms to curtail pathogen access to Zn^2+^. Despite our knowledge of Zn homeostasis, the manner in which the innate system modulates Zn^2+^ regulatory proteins in the context of fungal interactions and its influence on survival has been sparingly investigated.

**Figure 2:**
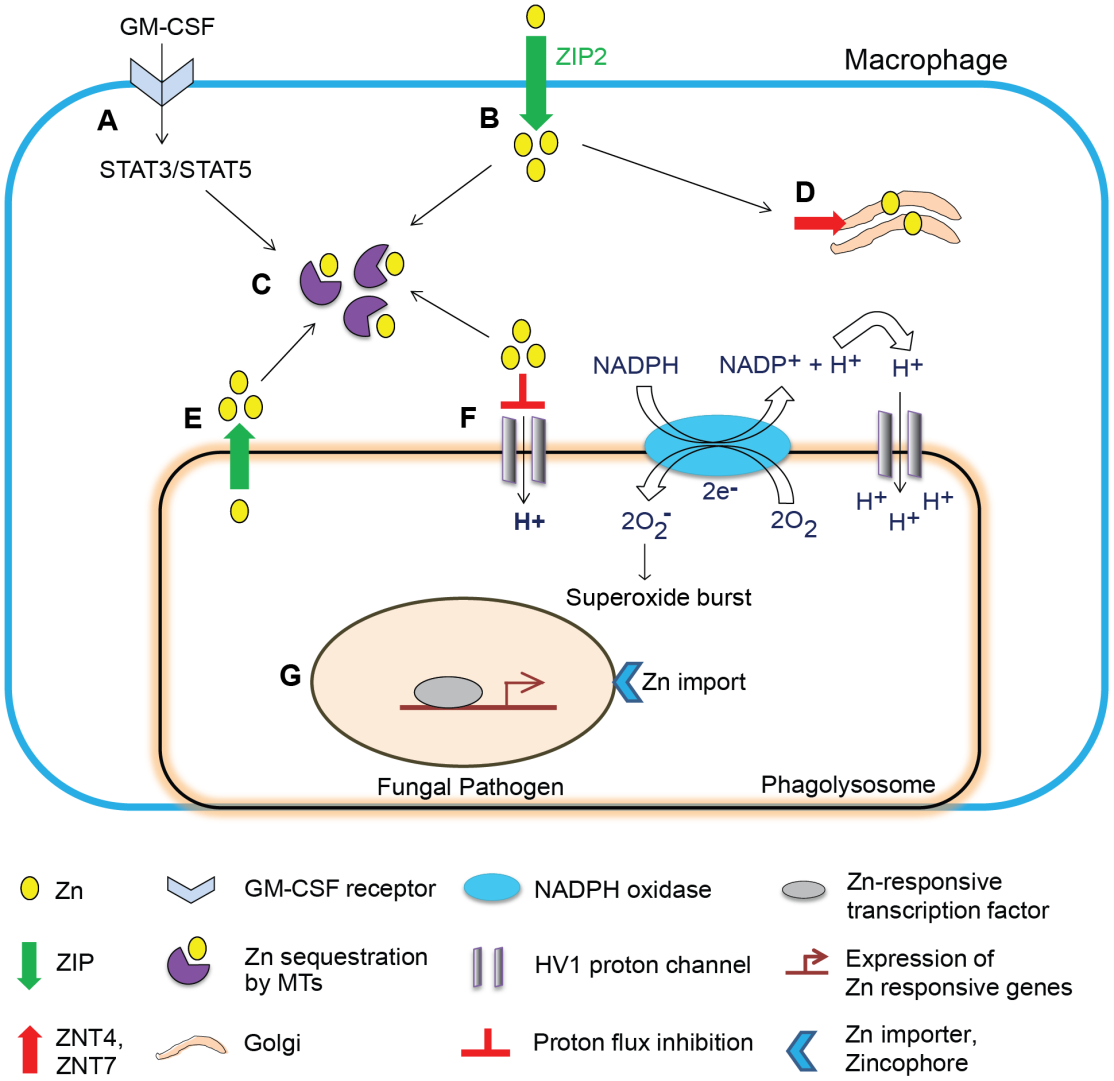
Schematic of Zn regulation in activated macrophages infected with a fungal pathogen. Zn regulation in a GM-CSF–activated macrophage leading to defense against fungal infection. (**A**) GM-CSF binds to the GM-CSF receptor on infected macrophages, activates STAT3 and STAT5 signaling, and triggers transcriptional activation in the nucleus. (**B**) Induction of ZIP2 causes increased Zn^2+^ influx, which may support increased metabolic functions to cope with stress in the infected macrophage. (**C**) STAT3 and STAT5 induce expression of MTs that sequester labile intracellular Zn^2+^. (**D**) Zn^2+^ is mobilized into the Golgi apparatus, associated with increased expression of Golgi membrane transporters ZNT4 and ZNT7. (**E**) Speculated lysosomal Zn deprivation by influx into the cytosol by ZIPs; the dotted arrow represents predicted sequestration of Zn^2+^ from this source by MTs. (**F**) Zn^2+^ inhibits proton flux via HV1, but the “Zn^2+^-deprived” environment lifts the inhibitory action (shown on extreme right of the phagolysosomal membrane) and H^+^ generated by Nox activity is channeled into phagolysosomes effectively sustaining production of superoxide radicals by the enzyme. (**G**) The pathogen senses a Zn^2+^-deprived environment and activates Zn-responsive transcription machinery to trigger Zn^2+^ import via fungal transporters and zincophore systems; ultimately, deficiency of Zn^2+^ starves the pathogen of this metal and simultaneously enhances superoxide burst in phagocytes, culminating into inhibition of fungal growth.

In macrophages, a dual stimulus involving GM-CSF and *H. capsulatum* infection potently induces Zn^2+^ influx by ZIP2. The enhanced Zn^2+^ uptake may reflect at least two possibilities: i) a stress response during infection to support macrophage functions such as increased transcription, and ii) a mechanism to deprive extracellular yeasts of Zn^2+^ analogous to the induction of hypozincemia during bacterial sepsis. This phenomenon can be viewed as an opportunity for *H. capsulatum* to exploit Zn^2+^ elevation to capture more labile Zn^2+^ within the host. However, despite increased influx, GM-CSF creates a state of “deprived” intracellular Zn^2+^ by two mechanisms. First, GM-CSF causes Zn^2+^ localization in the Golgi, a shift associated with expression of exporters ZNT4 and ZNT7 that export cytosolic Zn^2+^ into the Golgi. Second, signaling via signal transducer and activator of transcription STAT3 and STAT5 triggers the production of MTs that constrict the labile Zn^2+^ pool by binding the metal [18]. These studies highlight a fundamental Zn^2+^ sequestration property of transporters and MTs, which starves the pathogen and orchestrates a Zn^2+^ deprivation mechanism deployed by GM-CSF in macrophages **(** **Figure 2** **)**.

Spatial localization of Zn^2+^ transporters in the host potentially influences Zn^2+^ acquisition by intracellular fungi. ZNT4 enhances endosomal Zn^2+^ [27] that may be advantageous to the pathogen upon phagolysosomal fusion. In contrast, ZIP8 in T cells deprives lysosomal Zn^2+^ by importing it into the cytosol [28]. Though a role for importers in lysosomal Zn^2+^ deprivation has not been described in phagocytes, the existence of such a mechanism would starve the pathogen of Zn^2+^. Subcellular localization of transporters may be sensitive to the cellular microenvironment causing differential transport of Zn^2+^ in response to varying stimuli.

Apart from transporters and MTs, phagocytes produce calprotectin that binds Zn^2+^ with nanomolar affinity. Calprotectin in neutrophil extracellular traps (NETs) inhibits growth of *C. albicans* and also contributes to the fungistatic effect of plasmacytoid dendritic cells in *A. fumigatus* infection [29], [30]. Conversely, an inability to regulate excess of Zn^2+^ jeopardizes pathogen survival. In exploiting this to the host's advantage, human macrophages impose Zn^2+^ intoxication in mycobacteria-laden phagosomes. Mycobacterial P1-type ATPases efflux the metal and alleviate Zn^2+^ poisoning [31]. A role for heavy metal efflux pumps in fungal resistance to metal poisoning remains to be dissected. Thus, the immune system utilizes divergent Zn^2+^ restriction or intoxication mechanisms to combat infection. How immune cells preferentially utilize opposing stratagems against different microbial classes and the molecular cues that govern these decisions remain unclear. Collectively, Zn^2+^ modulation is a compelling arm of the innate system in restraining fungal persistence.

## Zinc Regulation: An Impact beyond Nutritional Immunity

Regulation of Zn^2+^ shapes the functional attributes of innate defense, impacting phagocyte function beyond nutritional immunity. GM-CSF–activated macrophages counter pathogen attack by eliciting a dual defense strategy comprising Zn^2+^ restriction to *H. capsulatum*, while concurrently enhancing phagocyte effector function. Zn^2+^ abates superoxide production by NADPH oxidase (Nox) by inhibiting hydrogen voltage-gated channel HV1. Fungi scavenge superoxide radicals via Zn and Cu or Mn dependent SODs [11], [12]. In activated macrophages, MTs bind Zn^2+^ and create an environment deficient in Zn^2+^ ions, in effect, sustaining HV1 and Nox function **(** **Figure 2** **)**. In this milieu, *H. capsulatum* is susceptible to ROS [18], presumably due to an ineffectual Zn and Cu dependent SOD response. The extent of Zn^2+^ deprivation by MTs results in effective superoxide production, and may simultaneously compromise fungal SOD-mediated defenses.

Collectively, Zn^2+^ restriction drives antifungal defense through a concurrent twofold effect: first, it induces Zn^2+^ starvation in the pathogen, and second, it strengthens oxidative burst-mediated defenses of the innate system. Thus, innate immunity is equipped with a variety of Zn^2+^ restriction strategies that function cooperatively to eliminate pathogens. As novel roles for metals are being described in dictating the outcome of immune regulation, we may be only beginning to appreciate the prominence of trace metals in immune defenses against infection.
